# High resolution seafloor thermometry for internal wave and upwelling monitoring using Distributed Acoustic Sensing

**DOI:** 10.1038/s41598-023-44635-0

**Published:** 2023-10-14

**Authors:** Julián David Pelaez Quiñones, Anthony Sladen, Aurelien Ponte, Itzhak Lior, Jean-Paul Ampuero, Diane Rivet, Samuel Meulé, Frédéric Bouchette, Ivane Pairaud, Paschal Coyle

**Affiliations:** 1 Université Côte d’Azur, CNRS, Observatoire de la Côte d’Azur, IRD, Géoazur, Sophia Antipolis, 250 rue Albert Einstein, 06560 Valbonne, France; 2 IFREMER, Université de Brest, CNRS, IRD, Laboratoire d’Océanographie Physique et Spatiale, IUEM, Brest, France; 3 Institute of Earth Sciences, The Hebrew University, Jerusalem, Israel; 4 Aix-Marseille Université, CNRS, IRD, INRAE, CEREGE, Aix-en-Provence, France; 5 Geosciences-M/GLADYS, Université de Montpellier, CNRS, Montpellier, France; 6 Aix-Marseille Université, CNRS/IN2P3, CPPM, Marseille, France

**Keywords:** Fibre optics and optical communications, Geophysics, Physical oceanography

## Abstract

Temperature is an essential oceanographic variable (EOV) that still today remains coarsely resolved below the surface and near the seafloor. Here, we gather evidence to confirm that Distributed Acoustic Sensing (DAS) technology can convert tens of kilometer-long seafloor fiber-optic telecommunication cables into dense arrays of temperature anomaly sensors having millikelvin (mK) sensitivity, thus allowing to monitor oceanic processes such as internal waves and upwelling with unprecedented detail. Notably, we report high-resolution observations of highly coherent near-inertial and super-inertial internal waves in the NW Mediterranean sea, offshore of Toulon, France, having spatial extents of a few kilometers and producing maximum thermal anomalies of more than 5 K at maximum absolute rates of more than 1 K/h. We validate our observations with in-situ oceanographic sensors and an alternative optical fiber sensing technology. Currently, DAS only provides temperature changes estimates, however practical solutions are outlined to obtain continuous absolute temperature measurements with DAS at the seafloor. Our observations grant key advantages to DAS over established temperature sensors, showing its transformative potential for the description of seafloor temperature fluctuations over an extended range of spatial and temporal scales, as well as for the understanding of the evolution of the ocean in a broad sense (e.g. physical and ecological). Diverse ocean-oriented fields could benefit from the potential applications of this fast-developing technology.

## Introduction

### Relevance of ocean temperature variability and experimental challenges

Monitoring seafloor ocean temperature variability became a priority over the last years within the Oceanographic community^1,2^. On climatic timescales, deep temperature measurements are needed to constrain the global ocean heat content and imbalance^3^, to monitor the evolution of water masses on regional scales^4^, climate changes^5^ and to predict the chemical^6^ and biological^7^ evolution of the ocean. Improved seafloor measurements within the coastal domain are much needed given their poor representation in climatic models^8^. At timescales of hours to minutes, ocean temperature variability is related to the internal wavefield and bottom boundary turbulent activity which affect the circulation of nutriments in the nearshore available to sustain marine life^9,10^, the propagation of hydroacoustic waves^11^ and the vertical mixing of the ocean at different scales^12^. The bottom boundary layer dynamics also remains an area of forefront research in both the coastal domain^13,14^ and the abyss^15,16^.

In-situ ocean thermometry typically relies on scattered point measurements and temporary deployments near the water surface (e.g., ships with thermosalinographs, buoys), which tend to be limited in terms of temporal and/or spatial resolution, while access to the deep ocean and remote regions remains challenging. Oceanographic moorings, Autonomous Underwater Vehicles, i.a. are attempting to fill this gap. However, obtaining a wide spatial coverage and long-term continuous measurements below the water surface and near the seafloor remains difficult^17^.

### DAS thermometry

In recent years, efforts have been devoted to transform fiber-optic cables into dense arrays of sensors with technologies that leverage various back-scattering effects of light pulses^18,19^. Among these, Distributed Acoustic Sensing (DAS) has gained wide interest thanks to its ability to monitor seismo-acoustic signals and dynamic strain with high sensitivity^20,21^, making it suitable for a wide range of geophysical monitoring applications^22–32^. Specifically, DAS systems rely on the analysis of the Rayleigh back-scattering spectrum of light. For some basics on DAS working principles, the reader is referred to the Supplementary Text S1.

The possibility of using Rayleigh-scattering systems to measure temperature changes had been recognized decades ago^33,34^. However, only in recent years there have been publications demonstrating the diverse geophysical applications of low frequency DAS (LF-DAS) signals that are dominated by temperature^35–40^. In particular, Ide et al.^38^ found distinctive patterns with several-hours periodicity on underwater LF-DAS data from a cable offshore Japan. They proposed that these patterns were related to the thermal signature of ocean water currents and their interaction with tides and complex bathymetric features. Lindsey et al.^22^ had also speculated about possible internal waves (IWs) signatures on LF-DAS data collected offshore California, USA. In practice, however, the nature of the physical signatures contained in underwater LF-DAS data is not entirely understood because of the lack of ground-truth validation and in-depth description of such signals.

From a theoretical perspective, the fluctuations in both the mechanical strain and temperature fields can locally change the optical path length^41^ of the fiber sensed by DAS interrogators^42–45^. At short timescales (${\lesssim }100$ s), DAS is expected to record mostly strain signals, since ambient temperature usually fluctuates much more slowly. At longer timescales, temperature fluctuations start to dominate over strain, mainly due to the contributions of the thermo-optic effect, that is, local changes in the refractive index of the optical fiber driven by temperature, while only to a minor extent by thermal expansion/contraction of the fiber^42,46–48^. Additionally, mismatches between the thermal expansion coefficients of the glass fiber and its host structure (e.g. sediments, concrete), or between the fiber and the cable jacket and/or steel armor, are prone to cause thermal stresses on the fiber^36,49^.

The two principal Distributed Fiber Optic Sensing (DFOS) technologies alternative to DAS are Distributed Temperature Sensing (DTS) and Distributed Strain and Temperature Sensing (DSTS). As stated by their names, both techniques are naturally designed for temperature monitoring. DTS and DSTS track variations in the Raman and Brillouin back-scattered spectrum of light, respectively^43^. A single DFOS system acts as a dense, gauge-adjustable array of point sensors along the cable layout, and thus measures a curvilinear projection of 3D physical fields. Previous studies relied on DTS to measure different underwater environments including lake and near-coastal seafloor temperatures, some of them describing internal waves, thermal events and tidal currents^50–56^. While DTS is dedicated to absolute temperature sensing, LF-DAS could provide a significantly higher sensitivity, spatio-temporal resolution and, potentially, a considerably longer sensing range for the measurement of temperature anomalies. As we will explore latter, both instruments can be considered complementary for physical oceanography applications.

In this study, we analyse LF-DAS (${\lesssim } 1$ mHz) signals on a seafloor telecommunication cable in the South of France and compare our results with independent ocean temperature measurements and DSTS data. We show that the recorded anomalies correlate with IWs and upwelling events, and are mainly, if not fully, related to temperature effects. Key applications of this technology for ocean monitoring in various contexts are discussed as well.

## Data

### Low-frequency DAS

**Figure 1:**
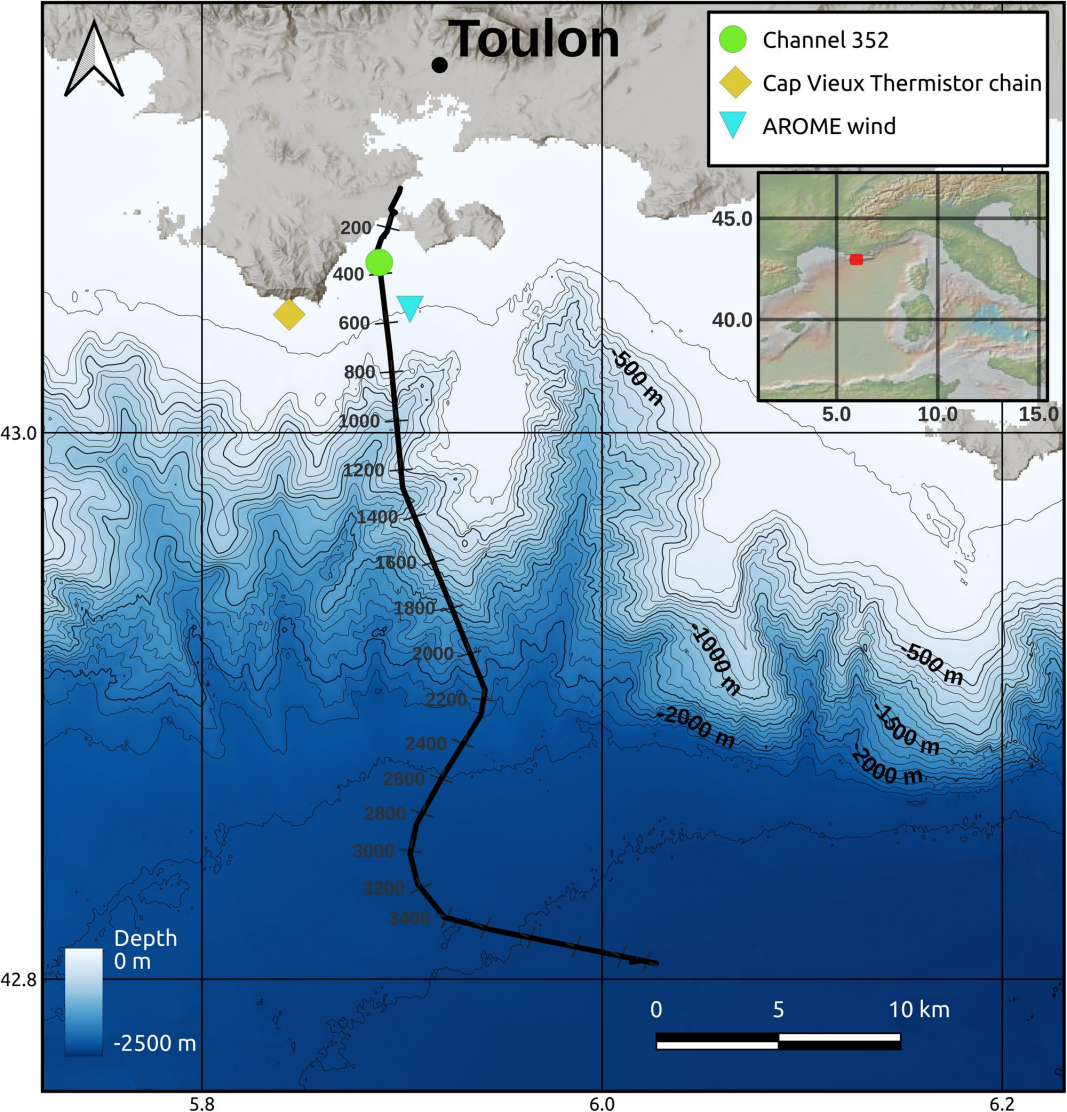
LSPM seafloor cable layout (black curve; numbered channels indicated) in the NW Mediterranean sea, south of Toulon. Bathymetry obtained from the Naval Hydrographic and Oceanographic Service of France (SHOM)^129^. In the following section, the temperature data of the thermistor chain (yellow diamond) is compared to channel 352 at $\sim$39 m depth (green dot) of the cable. Data of the AROME wind model are extracted at the position of the blue inverted triangle.

Our analysis focuses on nearly two weeks of data of a DAS campaign operated on July 2019 on a mostly exposed seafloor cable extending almost 45 km from Toulon, France, towards the NW Mediterranean basin, East of the Gulf of Lions (Fig. 1). In previous studies, this cable had been named MEUST-NumerEnv but is now part of the Laboratoire Sous-marin Provence Méditerranée (LSPM). We will therefore refer to it as the LSPM cable in the rest of the text. The data were acquired with a phase-sensitive Optical Time-Domain Reflectometry ($\phi$-OTDR) chirped-pulse DAS acquisition system^57,58^, providing strain measurements with both, spatial sampling and gauge length at 10 m. For a complete description of the acquisitions, see the “Methods” section. Our LF-DAS and DSTS observations are expected to remain mostly unaffected by low-frequency ($f \lesssim$ 1 mHz) strain signals, as the fiber is loose inside the cable, meaning that it can slide (creep) in the event that the rigid cable was slowly deformed^59^.

To isolate the low-frequency content of the large DAS dataset (11 Terabytes) and make it manageable for signal processing in a standard workstation, we applied a temporal moving average on the strain time series of each channel independently. Then, to convert the LF-DAS apparent strain values into corresponding absolute temperature anomalies (variations), we used the approximation: $\textrm{d} \varepsilon /\textrm{d} T = n\alpha ~+~\textrm{d} n/\textrm{d} T$^38,42,46,47^, where where $\varepsilon$ is the (apparent) strain recorded by DAS, *T* is temperature, *n* the optical fiber refractive index and $\alpha$ its thermal expansion coefficient. This conversion, as well as the full data pre-processing scheme, are detailed in the “Methods” section.

### Oceanographic and meteorological data

Our validation of the LF-DAS measurements relies on temperature observations collected along a vertical thermistor chain of 10 sensors (5 to 50 m depths) off Cap Vieux, Toulon (Fig. 1) recording every half-hour at $\pm 0.2^\circ$C accuracy^60^. The deepest sensor is only a few centimeters above the seabed. The thermistor chain is located over the gently sloping shelf south of Toulon, about 4 km west of the closest cable section^61,62^ (see “Methods” section for additional technical aspects.)

Additionally, hourly wind data (horizontal speed components at 10 m-height and turbulent surface stresses) of Météo-France operational forecasting atmospheric model AROME^63^ near the LSPM cable is used to assess the potential relationship between wind events and LF-DAS. The spatial grid of this model is of 0.01$^{\circ }$ ($\sim$1.3 km). Wind station data were not available near the cable.

## Results

### LF-DAS variability—time series

#### Variability on multiple days timescales

**Figure 2:**
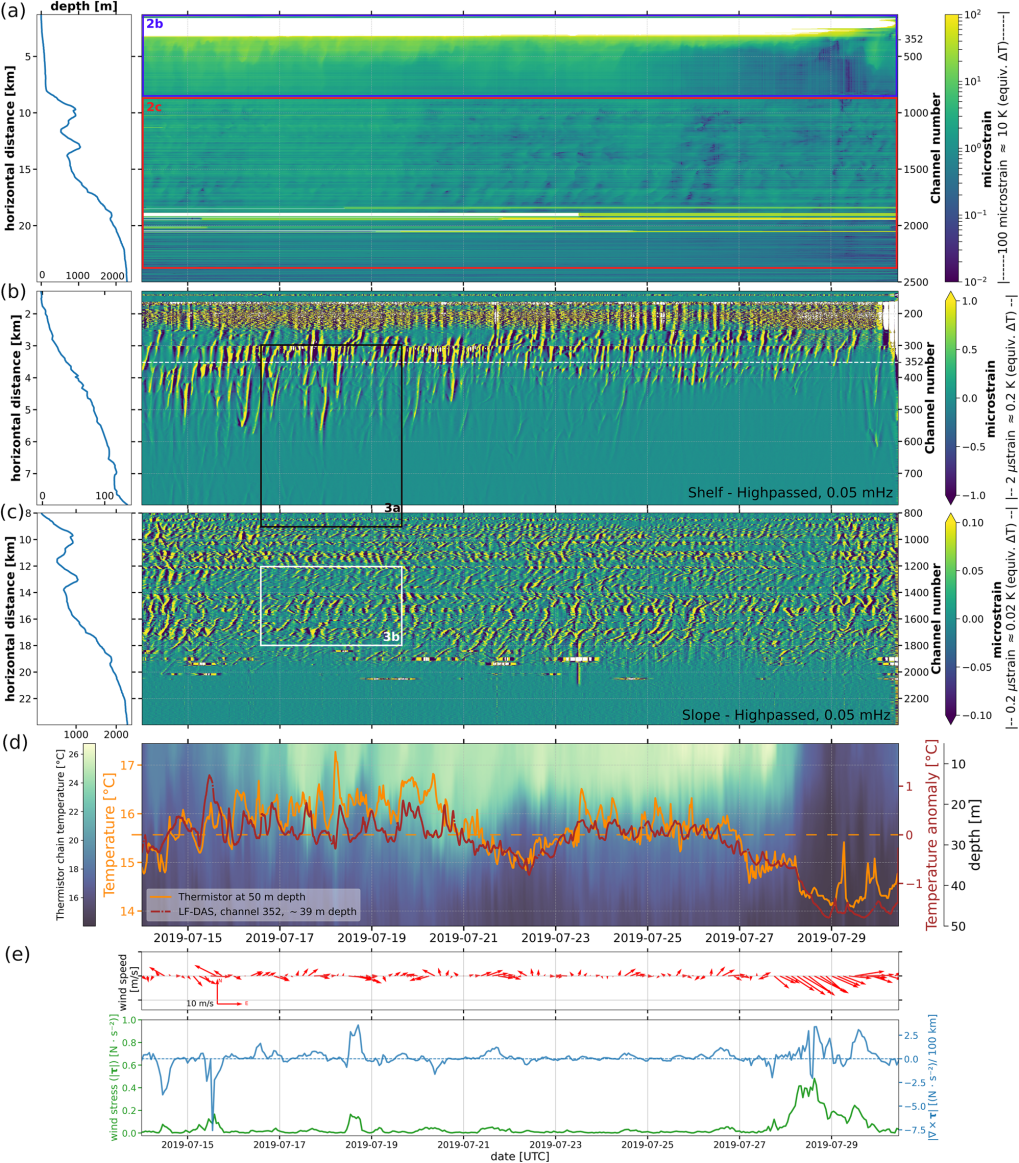
July 2019 LF-DAS data on the LSPM cable and reference ground truth time series. (**a**) Raw LF-DAS section from the shoreline to the deep Mediterranean sea along with bathymetry along the cable (left). Values outside the colorbars (with estimated equivalent temperature variation range on the far right) are clipped. Purple and red frames correspond to the same ranges of (**b**,**c**). Highpass-filtered subsets (0.05 mHz, 3-pole Butterworth) along (b) the continental shelf (with max/min-saturated colorscale, clipped at ±5 microstrain) and (**c**) slope/rise subsections (saturated colorscale, clipped at ±1 microstrain). White and black frames correspond to the same ranges used in Fig. 3. (**d**) LF-DAS channel 352 (identified with a horizontal dashed line in (**b**)) converted to estimated absolute temperature differences (in red, scale on right side) relative to the mean temperature (dashed line) of the 50 m-depth thermistor sensor (in orange, scale on the left). Grid spacing is shared for both curves. The colormesh of vertical temperature distribution at the thermistor chain in Cap Vieux (with depth scale in the far right) depicts the water column layering evolution. (**e**) AROME horizontal wind azimuth vectors (above), total wind stress (|$\tau$|, below, in green) and stress curl magnitude (|$\nabla \times \tau$|, below, in blue). All figures share time span.

Figure 2 summarizes our LF-DAS observations. Only the first 25 km of the LSPM cable (from the shoreline to the continental rise) are shown, given that our data has a significantly lower signal-to-noise ratio (SNR) at longer ranges. The evolution of apparent strain values of LF-DAS in the time-range space (Fig. 2a) indicates that the largest variability on multiple days timescales is found over the continental shelf (within 100 m water depths). This is consistent with the larger thermal stratification in the upper ocean expected in general and observed in the study area (Fig. 2d). LF-DAS values corresponding to equivalent temperature differences exceeding 10 K are not plotted in Fig. 2a, as these are considered too large for typical ocean temperature variability and are presumed to be partially biased by coastal wave activity, e.g. surface gravity wave-induced stresses or nonoptimal seafloor coupling. For instance, the first $\sim$500 m of cable are known to be mostly buried, after which the cable remains mostly exposed, which is supported by observing that LF signals are virtually non-existent for most of the first few hundreds of meters of cable (Fig. 2b). Temperature differences observed in the shelf often exceed 1 K near the shore and can reach up to 5 K (purple box in Fig. 2a), while the slope and deep water section (red box in Fig. 2a) mostly contains thermal oscillations below 1 K. Figure 2b,c are high-passed versions of the coloured regions indicated in Fig. 2a for better visualization of the structure of the anomalies (discussed in the following subsection). For the thermistor chain, the root mean square (RMS) temperature values at its deepest sensor (50 m) were 0.39 and 0.25 K at sub- and superinertial/inertial bands (below and above 0.005 mHz), respectively, while the maximum absolute values on the same frequency bands reached 0.64 and 1.11 K, respectively (peak-to-peak values of up to about 1.2 and 2.2 K).

The multiple-day temperature signal recorded at the Cap Vieux thermistor chain correlates well with the LF-DAS signal at the best-matching channel (Fig. 2d), having a zero-lag Pearson correlation coefficient of 0.82. This channel was identified via maximum cross-correlation search (additional details in “Methods” section) and lies approximately on the 39 m isobath, which is comparable to that of the Cap Vieux sensor at 50 m depth, located at the seafloor as well. The lower frequency trend of the LF-DAS signal at the best matching channel contains peak-to-peak temperature fluctuations between 1 to 2 K, while the observed RMS (root mean square) values are 0.30 and 0.19 K below and above 0.005 mHz, respectively. The magnitude of these temperature changes is thus comparable to those of the deepest sensor of the thermistor chain, as evident from Fig. 2d. A major cooling event towards the last days of the DAS campaign is clearly reflected in the temperature in situ and LF-DAS observations that coincides with an intense (high wind stress magnitude) northwesterly wind event lasting a few days and having predominantly cyclonic horizontal wind stress curl magnitude (> 0 in the northern hemisphere^64,65^), as attested by the AROME data (Fig. 2e).

#### Variability on multiple hours timescales

At hourly-to-daily scales, a highly variable spatial extent and propagation character of the LF-DAS signal (Fig. 2b,c) and its rough waveforms (characteristic edginess, sharp onsets and decays, Fig. 2d) are evidenced. Over the shelf for instance, a progressive retreat of this high frequency variability towards the shore throughout the experiment stands out (Fig. 2b) which may indicate a several-day evolution of the regional thermal stratification and is consistent with its observed decrease at Cap Vieux after the 23rd of July (Fig. 2d). The thermal oscillations are persistent from the shallow-most continental shelf down to almost the bottom of the continental slope at 2000 m depth. In the deep sea region (beyond about channel 2000, at $\sim$1.8 km depth), the data suggests a thermally stable area with temperature variability close to or below the optical noise threshold of the implemented DAS unit. From Fig. 2b,c it can be seen that at the shelf, the apparent cross-shore extent of single anomalies (where the cable layout is nearly horizontal) range between a few hundred meters and up to 4 km, while at the slope, the along-cable scale of single anomalies can cover 1$\sim$2 km, although here assessments are highly apparent because of the non-monotonic, steep bathymetry.

**Figure 3:**
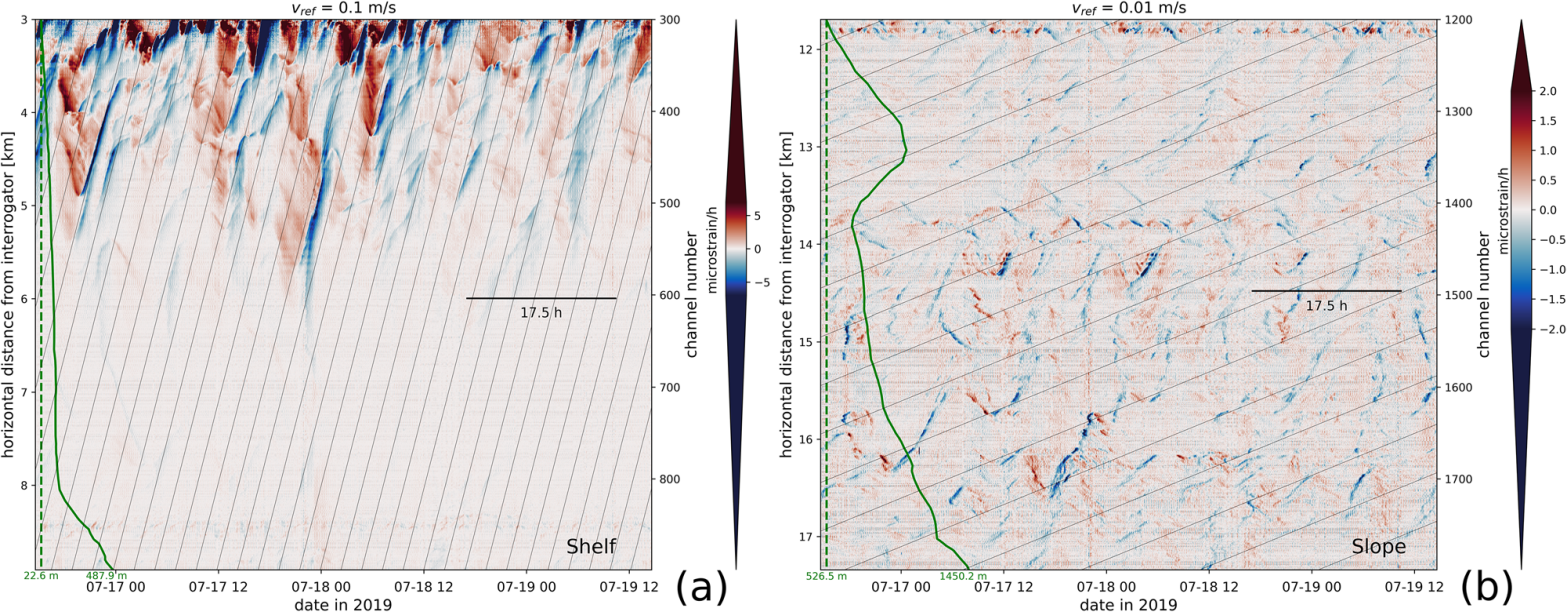
A time-differentiated subset of the July 2019 LF-DAS data on the LSPM cable shown in Fig. 2 for the continental shelf (**a**) and the continental slope (**b**), both covering the same four days of data and sharing an equal number of channels. Reference propagation speeds (thin, slanted lines) are indicated on the (top) heading of each sub-figure. Green curves depict corresponding bathymetry along the cable (same scale in both plots) with the minimum (dashed line) and maximum depths indicated. The theoretical inertial period of the study region ($T_c\approx$17.5 h) is indicated with a horizontal line. The saturated color regions (extended triangles) of the linear colorbars are proportional in length to their rectangular interiors.

Hourly-to-daily fluctuations of LF-DAS on channel 352 exhibit some visual similarity with those of the Cap Vieux temperature in amplitude, shape and periodicity (Fig. 2d). However, the exact waveforms and phases differ at each location and both time series are as a result only roughly correlated at these shorter timescales (maximum Pearson correlation coefficient at 0.20, depending on moving average trend removal parameters, see “Methods” for details), which may be explained by the fact that the spatial scales associated with these fluctuations are smaller than the cable-thermistor chain separation. In general, the intermittent LF-DAS temperature arrivals (anomalies with slanted time-space offsets) over the continental shelf (Fig. 2b) and slope (Fig. 2c) indicate locally coherent propagation in both, on- and offshore directions. LF-DAS signals are composed of abrupt fluctuations that rapidly rise (warm) and decay (cool) back to a baseline level (Figs. 2d and 3). Along the continental slope, oscillations are more ubiquitous and repetitive over time than those at the shelf. In both, the continental shelf and slope, the fast oscillations reveal the complex, fine scale variability of the LF-DAS signal as well as its high sensitivity to small temperature changes ($\lesssim$ 1 mK).

A detailed view of the data presented in Fig. 2 can be found in Fig. 3. LF-DAS observations are differentiated in time to sharpen the image and highlight fast variability. Along the continental shelf section (Fig. 3a), single bore-like features reaching absolute temperature fluctuation rates of more than 6 microstrain/h (nearly equivalent to 0.6 K/h) dominate and occasionally reach more than 1 K/h. These mainly consist of persistent “V”-shaped anomalies with variable spatial scales and unequally distributed in time and space, although mostly clustered over the shallow section of the shelf, between about 10 to 60 m depths. A dominant, onshore apparent speed component close to 0.1 m/s is evident. Notably, the offshore propagating anomalies are mostly slow warming events, while faster cooling events dominate the onshore component.

At the continental slope section (Fig. 3b), repetitive oscillations with absolute temperature change rates mostly equivalent to less than about 1 microstrain/h, i.e. 0.1 K/h, but reaching more than 0.2 K/h, are observed that are smaller than those at the shelf. A broader distribution of apparent speeds is also evident, the slowest reaching $\sim$0.01 m/s. A visible along-channel modulation of the LF-DAS patterns (amplitude and phase propagation) suggests a marked site effect modulation, potentially related to the changing water depth, bathymetric slope or variable cable-seabed coupling, burial degree and/or cable orientation. Features on the onshore-descending flank of the valley at 13 km from the interrogator appear to propagate in the opposite sense to those on the facing flank and the rest of the continental slope (see the reversal of “V-shaped” patterns in Fig. 3b), indicating a bathymetric slope control in the orientation of the anomalies. The thermal oscillations are also generally weaker across this valley.

### LF-DAS variability—spectra

The relatively short time span of the data hampers a Fourier-derived spectrogram that properly resolves LF signals in time. Furthermore, the widespread sharp patterns of the LF-DAS time series affect the reliability of the finite Fourier Transform. In order to overcome these obstacles, we conduct an Empirical Mode Decomposition (EMD) analysis^66–70^ based on the Hilbert-Huang transform (HHT)^71^ which is intended for decomposition of non-linear and non-stationary signals. Supplementary text S3 describes details on the parameterization of the EMD and HHT.

**Figure 4:**
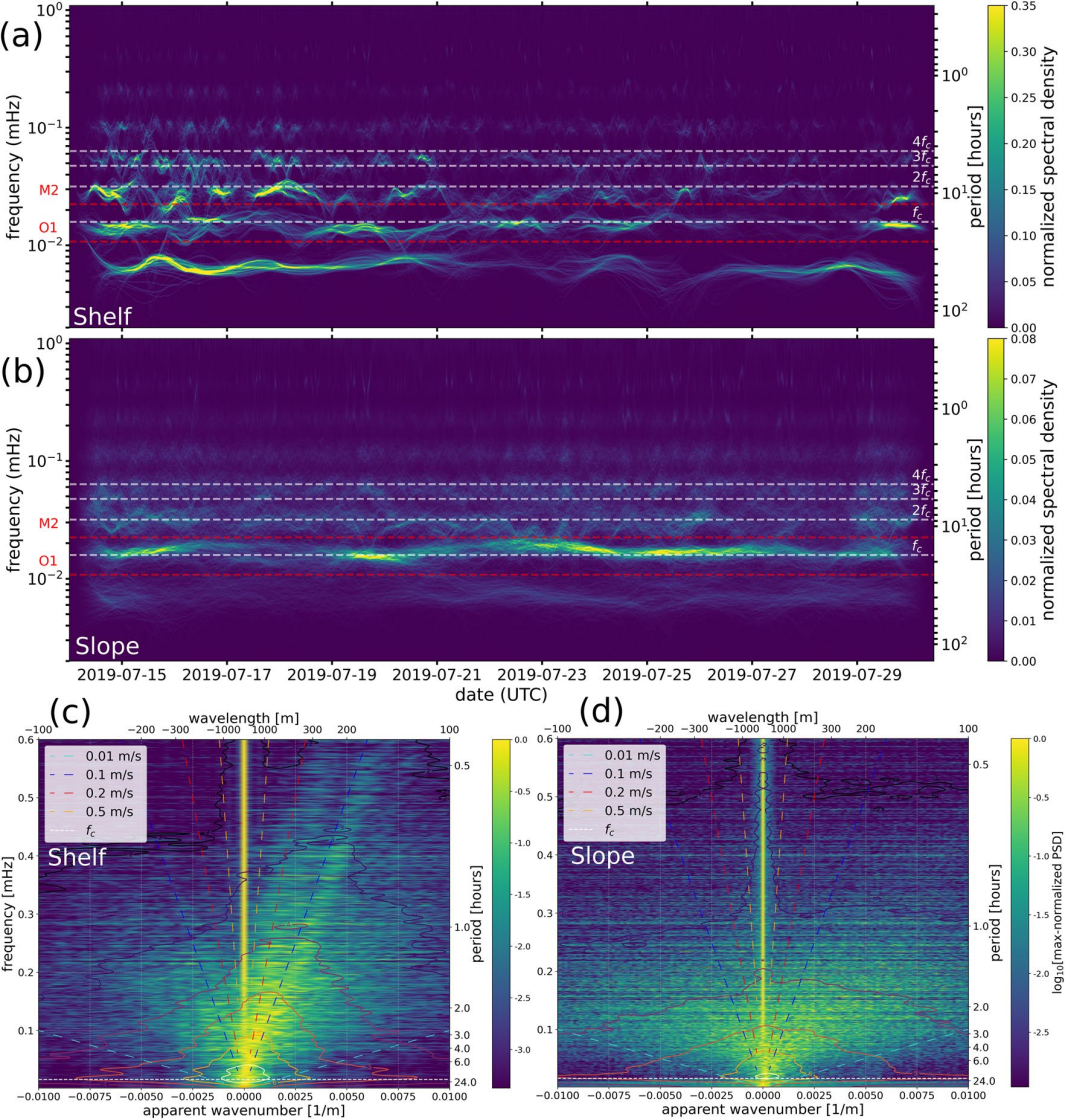
July 2019 LF-DAS spectra (same time span as in Fig. 2) on the LSPM cable. Average Hilbert-Huang spectra with tapered edges for the shelf (b) and slope (c) cable sections. Frequency-wavenumber spectra (max-normalized across frequency) in colormap with its raw values indicated in colored contours ($\log _{10}$[microstrain$^{2}\cdot$m/Hz] units) for the shelf (d) and slope (e) cable sections. The inertial frequency $f_c=T_c^{-1}$, its first three expected harmonics and the $O_1$ and $M_2$ tidal components are marked with colored dashed lines. Reference speeds (dashed, sloping lines) are indicated in the frequency-wavenumber spectra.

Figure 4a,b show the results of averaging the instantaneous frequencies of each of the EMD Intrinsic Mode Functions (IMFs, see Supplementary Text S3 and Fig. S1) obtained for each channel across the shelf (channels 350-800, from 37 to 138 m depth) and slope (channels 800-2000, from 138 to 1870 m depths) cable sections, respectively. There is a clear modulation of the energy over time for both the shelf and slope sections. The spectral energy distribution over the shelf area (Fig. 4a) indicates a rich spectrum of motions with periods ranging from multiple days to multiple hours having sporadic transient events and a comparatively more non-stationary character than the steadier signal over the slope, as expected from the time series signatures. Over the slope (Fig. 4b), variability correlates well with the expected inertial period in the study region, $T_c=f_{c}^{-1}{\approx }17.5$ h (indicated visually in Fig. 3) and potentially with some of its first higher-order harmonics, as suggested by the persistent and well-defined spectral energy bands (Fig. 4b). $f_c$ refers to the latitude-dependent Coriolis parameter (further details on inertial variability in Supplementary text S2). This is indicative of near-inertial internal waves (IWs). As expected from the microtidal character of the Mediterranean sea, the spectral energy peaks are not well correlated with the main tidal components but instead to inertial oscillations, as previously observed in the region^72–75^.

Figure 4c,d depict frequency-wavenumber^76^ spectra on continental shelf (channels 400-800, depths 55-138 m) and slope (channels 1100-1800, depths 730-1452 m) sections where the horizontal projection of the LSPM cable is nearly linear. As expected, low-frequency signals approaching the inertial peak dominate both spectra, as illustrated by the contours. Onshore propagation components (positive apparent wavenumbers) over the continental shelf (Fig. 4c) are more prominent than those offshore. Here, the onshore thermal component contains at least two coherent, non-dispersive arrivals at apparent speeds between about 0.1 and 0.2 m/s that suggest modal propagation. A similar coherent component at 0.1 m/s can be faintly seen in the offshore spectrum. These speeds compare well with the observations of Fig. 3 and remain within the range of typical ocean current speeds in the ocean. The same asymmetry is less so clear over the slope (Fig. 4d), where the offshore component is only slightly weaker and the frequency-wavenumber spectrum is more smeared. This may arise from several factors, including the more irregular bathymetry affecting the cable layout at the slope, the wide distribution of speeds and scales of the anomalies and the lower SNR. The apparent wavelengths of the dominant energy components range from about a couple hundreds of meters to several kilometers, in line with typical scales of IWs in the ocean^10,77^. Zero-wavenumber energy is dominated by residual optical common-mode noise.

## Discussion and perspectives

### Upwelling event and several-days temperature variability

A cooling event corresponding to an average estimated decrease of ${\sim }2$ K across the entire continental shelf ($\sim$8 km-wide) is evidenced towards the end of the LF-DAS observation period (Fig. 2a–e) which is consistent with coastal upwelling^78^ caused by northwesterly Mistral wind episodes in the region^79,80^, as confirmed by the high wind stress and its cyclonic curl, which enhaces upwelling through Ekman pumping^81,82^. The Cap Vieux (thermistor chain) temperature measurements independently confirmed this cooling event, which is associated with a homogenization of the water column temperature. Sea surface temperature estimates at 1.2 km-resolution provided by the operational model F2-MARS3D-MENOR1200^83,84^ also confirmed this upwelling event, which started on the 27/07/2019 at the northwestern-most tip of the Gulf of Lions and propagated towards the SE, in consistency with a Mistral-wind origin (see Supplementary Video S1, where the black square corresponds to the approximate location of the thermistor chain). As the studied shelf region is located in an well-known Mistral wind-driven upwelling area^85–87^, strong cooling of coastal waters in summer in the shelf off Provence are expected which could explain the observed temperature decreases of a few degrees in the neighborhood of the thermistor chain, at shallow ($\lesssim$ 20 m) depths. For comparison, more than 5 K decreases were reported during an upwelling event at 20 m depth in 2017, west of the Cassidaigne canyon, which only lies $\sim$30 km away from Cap Vieux^88^. The area is also influenced by general circulation, with mesoscale structures of the near-surface Liguro-Provençal (i.e. Northern) current bringing (generally) warmer water to the area over a large portion of the water column^89–92^, which in turn could potentially be related to the multiple-day modulations present in LF-DAS, as these could produce temperature changes over several days. This highlights the potential of LF-DAS for capturing the propagation characteristics of ocean seafloor variability on multiple days time scales and suggests the study of large-scale ocean currents, which are known to account for several-days temperature fluctuations near the bottom.

### Near-inertial and super-inertial temperature variability

The LF-DAS observations reported here are consistent with past observations and canonical theories of the oceanic internal wavefield in general^93^ and highlight the presence of near-inertial ($f^{-1} \sim$17 h) and super-inertial ($1\lesssim f^{-1}<17$ h) IWs having apparent wavelengths in the range of several hundreds of meters that produce maximum temperature fluctuations at the seafloor-water boundary of more than 5 K in the near-shore (down to about 20 m depths) and of less than 1 K over the continental slope (between about 200 to 2000 m depths). The signal over the deep sea (below 2000 m depths) is unclear and might have magnitudes at or below the sensitivity limit of LF-DAS.

#### The strain and temperature sensing transition of DAS

Weak temperature variability with periods of less than a couple hours to a few minutes ($\sim$0.1-1.0 mHz) is ubiquitous in the time series and the spectral analyses. For ocean-related processes, this spectral band is expected to be influenced by buoyancy forces in the ocean (i.e. internal gravity waves). Tides are typically very weak in the Mediterranean sea, and the lack of any clear tidal signal in our data suggests that their contribution in the study region is rather small. However, this may also correspond to the fact that the strength of internal tides is related to both, the strength of the (weak) Mediterranean surface tide and the barotropic tidal flow being forced across bathymetric variations (e.g. over sills or shelf breaks with wide shelves), in a way that the bathymetric configuration of the shelf (Figs. 1 and 3) does not favor strong tidal-driven seafloor temperature oscillations. This is supported by first-order estimates of the temperature fluctuations induced by tidal currents for a diurnal (24 h) period assuming a steady vertical temperature gradient, as outlined in Supplementary Text S4 and depicted in Supplementary Fig. S2. The RMS estimates are in the order of 0.1 and 0.01 K at the shelf and the continental slope, respectively, while the observed LF-DAS variability reached >1 K at the shelf and up to nearly 1 K over the continental slope. Thus, although generally smaller than the observed temperature anomalies by nearly an order of magnitude, tidal temperature anomalies are not expected to be entirely negligible. We note however, that the actual tidal temperature anomalies are expected to be even smaller than our estimates, mainly because of the implicit assumption of our model that tidal currents travel across isobaths, which is often not the case. Further studies are required to better understand the complex near-coastal thermal dynamics of the study region and its potential relationship with tides.

The detection of mechanical strains at tidal frequencies has only been demonstrated for DAS under laboratory conditions, although only for controlled deformations that were several orders of magnitude larger than actual tides^94^. Furthermore, DAS is also known to have a highly directional sensitivity pattern^20,95^, as the deformation response of optical fibers to tangential (broadside) stresses is generally expected to be much lower than longitudinal (axial) ones^96^, meaning that vertical pressure waves induced by tidal oscillations inciding a fiber in a gently sloping, flat bottom might go undetected. Furthermore, it is known that the response of DAS is generally inversely proportional to the apparent wavelengths generated by such broadside incidence angles^97–100^. On the other hand, the detection of horizontal seafloor motions induced by tides over cable sections with rugged or sloping bathymetry remains to be demonstrated for DAS.

The seismic hum^101^ is a well-known long-period strain signal that is generally expected to fade below about 2 mHz. However, it has been proposed, along with (the transient and intermittent) tectonic earthquakes, as a continuous forcing mechanism of some of the normal modes of the earth^102^, whose resonance frequencies partially overlap those of the several-minute period thermal variability here observed. As these normal modes could be continuously observed at the seafloor^103^, further analyses are required to quantify their potential contribution to LF-DAS in the form of long-period strain signals.

#### Internal waves on the continental shelf

The variable cross-shore extent of the shelf temperature variability of up to 4 km over time and depth can result from regional variations in the vertical thermal stratification of the ocean. Local changes in the magnitude of the thermal anomalies, reaching more than 5 K and absolute thermal rates of more than 1 K/h, might also arise from IW packets with variable amplitudes displacing the thermocline vertically. In general, shallower areas contain larger thermal anomalies than deeper regions, following the general vertical attenuation of IW away from the thermocline. These anomalies become scattered at the near-shore, as several signatures appear to accumulate and overlap, which is not surprising, as IWs are expected to degenerate considerably at the near-shore due to non-linear effects. The substantial reduction in high frequency variability over the course the 2019 experiment (Fig. 2b) is concomitant with the thermal homogenization of the water column induced by the upwelling event discussed before, as the amplitude of IW tends to be proportional to the sharpness of pycnoclines. This illustrates well the dependence of IW dynamics on temporal variations in the vertical stratification of the ocean.

The widespread “V”-shaped thermal signatures over the shelf resemble those of single, well-defined bores propagating on- and off-shore at nearly constant speeds. Lucas & Pinkel (2022)^56^ also observed similar patterns with DTS measurements in the near-shore and explained them in terms of vertical water oscillations induced by IWs that in turn advect the vertical water temperature gradient (e.g. the thermocline) against a gently sloping bottom. This would account for the “V” anomalies with slow warming and fast cooling events propagating in opposite senses (observed over both, the shelf and the slope). Interestingly, Lucas & Pinkel (2022) observed the same behavior at tidal frequencies, except for a reversed thermal rate asymmetry, meaning fast warming and slow cooling events. For our data, the control exerted by the direction of the slope relative to that of the anomalies (Fig. 3b) would suggest predominant IWs of depression propagating offshore and/or elevation waves propagating onshore. A simplified diagram schematizing the expected LF-DAS cooling/warming signatures for an along-slope monotonically-oscillating current (e.g. prompted by a low-mode internal wave) advecting a sharp thermocline is presented in Supplementary Fig. S3.

#### Internal waves on the continental slope

The lack of correspondence between the observed thermal signatures and atmospheric variability supports the presence of persistent near-inertial oscillations. Previous studies had documented energetic near-coastal inertial IWs in the of Gulf of Lions^72,104^ and the Western Mediterranean abyss^73^. The ubiquitous presence of near-inertial variability over the slope can be explained by the more stable ocean thermal stratification expected at these depths. Here, LF-DAS points towards persistent cold water anomalies propagating onshore, which may be of substantial relevance as these are known over the shelf^10^ but less so over the slope. The mean fluctuation amplitudes here are on the order of 0.01 K, while the thermal rates occasionally exceed 0.2 K/h. Assuming a vertical thermal stratification of $10^{-3}$ K/m, such amplitudes amount to vertical displacements of about 10 m and near-inertial vertical velocity amplitudes of $10^{-3}$ m/s. At the seafloor, horizontal and vertical velocities are tied via the bottom boundary condition: $w + {\textbf{u}}\cdot \nabla h=0$ where *w* and ${\textbf{u}}$ are the vertical and horizontal flows respectively, and *h* is water depth. Assuming an average slope of 0.1 (Fig. 2c), this leads to horizontal velocities of 0.01 m/s. This value is comparable to typical propagation speeds observed on Figs. 3 and 4c,d, and remains in line with past observations of IWs in the area^73^. Our analysis assumes a dominantly advective scenario, however, a diffusive component may exist that accounts for a fraction of the observed thermal propagation rates.

Our results are also compatible with IWs producing a dominant onshore temperature anomaly propagation component (Fig. 4c,d). The offshore energy could be partially comprised of horizontal reflections at bathymetric obstacles, as near-inertial IWs mostly reflect horizontally against slopping bottoms^105^. However, it is well-known that IW packets do not generally propagate horizontally. In fact, the deep inertial motion has an upward phase component and downward group propagation when stratification (*N*) is larger than $f_c$^106^. Both propagation vectors have equal-sign vertical components for gyroscopic IWs, that is, when $N{\approx }0$^107^. Taking into consideration the strong dependence of the observed IW on the continental slope bathymetry, the effect of the variable seafloor steepness and roughness has to be taken into account for a precise description of IW energy partitioning at the seafloor, including reverberations and higher order modes (e.g. Fig. 4c) produced at critical incidence^108^.

### LF-DAS and alternative DFOS approaches

**Figure 5:**
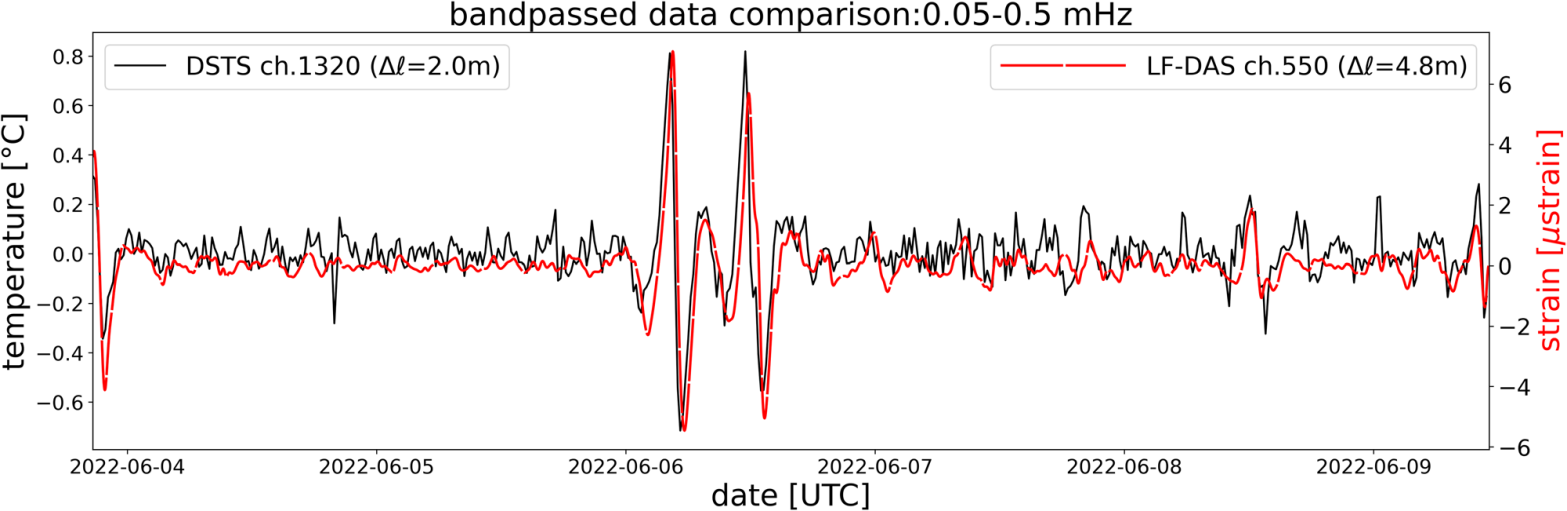
Comparison of simultaneous DSTS and LF-DAS measurements at collocated channels on parallel fibers of the LSPM cable, June 2022. Corresponding gauge lengths are indicated ($\Delta \ell$). Both signals are bandpassed in the 0.05-0.5 mHz range.

Standard DAS and DSTS systems cannot distinguish temperature or strain anomalies without external information on the processes involved (e.g. frequency or shape of the perturbation). However, at low frequencies, the temperature effect is expected to dominate, as evidenced by previous works and our validation in Fig. 2d. This key point is also supported by the independent acquisition of simultaneous DAS and DSTS on the LSPM cable. Figure 5 shows the LF-DAS and DSTS time series, bandpass-filtered from 0.05 to 0.5 mHz, a range where the frequency content of both instruments is comparable. Apart from some deviations in the weaker, fast fluctuations, LF-DAS matches the DSTS signal. The former appears smoother because of its longer spatial sampling (4.8 m for LF-DAS and 2.0 m for DSTS) and/or increased high frequency noise in the latter. Apparent time lags are likely related to the different spatial samplings of each deployment and the absence of clock synchronization. Visual inspection of Supplementary Fig. S4 confirms the similarity of both data types and that the DSTS signal has a lower SNR than LF-DAS at long ranges. Conversely, DSTS appears to have a higher SNR than LF-DAS near the shoreline, potentially due to the increased sensitivity of DAS to surface gravity wave strains and other near-coastal conditions affecting the signal.

Ide et al.^38^, related LF-DAS data acquired offshore Japan with temperature changes of a few Kelvins having apparent propagation speeds of $\sim$0.5 m/s. Our LF-DAS observations also confirm temperature changes of some Kelvins on the continental shelf, and others on the order of $\sim$0.1 K on the continental slope, both having similar apparent propagation speeds. Having in mind that standard fibers and DAS systems have sensitivities of the order of a nanostrain, LF-DAS measurements should be sensitive to temperature variations of at least $\sim$0.1 mK.

Upon calibration, DSTS and DTS are capable of providing absolute temperature measurements^109^, while LF-DAS is currently restricted to absolute temperature variations because of the $\phi$-OTDR limitations^44^. Yet, LF-DAS offers some key advantages for monitoring thermal anomalies: over short distances ($\sim$5 km), most DSTS and DTS interrogators typically have repeatability^43^ on the order of 0.1$\sim$1.0 K (also depending on type of fiber, duration of acquisition, environmental setting, i.a.), while LF-DAS approaches $\sim$0.1 mK. For DSTS and DTS, the repeatability drops sharply with the sensing range, e.g. $\sim$1.5 K at 70 km for a single-mode fiber with a minimum laser attenuation of 0.2 dB/km^110^. This also implies that, at long distances, DTS measurements need to be averaged over longer times (tens of minutes or more) and over larger gauge lengths to achieve acceptable performances^110^. In contrast, the Rayleigh scattered power is 20 to 30 dB higher than the Brillouin and Raman scatterings typically used for temperature sensing, respectively^111^, so that longer sensing ranges are attainable with DAS (up to 100 km and more^112^). At the same time, diverse techniques exist to preserve optimal DAS repeatability at long distances^21^.

### Challenges and limitations

The current lack of knowledge about the exact transfer function between the optical fiber response and the input ambient temperature hampers the exact estimation of the latter. Although this transfer function is reportedly linear^38,40^, it is generally expected to be a function of the composition and structure of each cable^36,113^ and its coupling and thermal insulation by the host medium, from which detailed information is often lacking. This limitation, however, could be overcome through unique, temporary or regular temperature calibrations at single or multiple cable locations with dedicated temperature sensors and/or with auxiliary DTS/DSTS systems^114^, depending on the required precision and possible logistics. When implemented, the SMART cable initiative^115^ should provide calibrated temperature sensors at the optical repeaters of new cables. It is also worth reminding the significant efforts and recent progress on the improvement of the sensing range, SNR characteristics and the simultaneous use of DAS instruments on operating telecommunication fibers^21,112,116–120^. Although standard DAS units are expected to suffer from noise increasing inversely proportional to frequency (1/f), our results show that this effect is not enough to impair the detection of the thermal oceanic signatures at the frequencies here considered. In a recent study^121^, the possibility to suppress the 1/f noise was demonstrated, thus opening the way for a new generation of DAS systems robust for static sensing and capable of providing absolute temperatures over periods of months or longer.

Currently, LF-DAS on a single cable only provides a one-dimensional view of the multi-dimensional oceanic variability, therefore more advanced wavefield processing methods (e.g. beamforming, correlation analyses) and additional constraints (e.g. multiple cables or additional ground truths) could provide further insights into the 3D IW propagation parameters, such as true wavelengths, shapes and speeds. Future studies may also address other interesting physical signals and effects potentially present on LF-DAS. For instance, variable hydrostatic or hydrodynamic pressure loads and coastal surface gravity wave-related stresses could exert an effect on the thermo-optic sensitivity of the cable. Also, the degree of cable burial under sediments is expected to bias the DAS sensitivity to water temperature anomalies due to thermal insulation and potentially delay the signal response. Local turbulence that influences the thermal signatures at shorter timescales may also exist. In active volcanic regions and others with geothermic or fluid injection activity, underground heat anomalies might as well be prone to monitoring with LF-DAS.

### Conclusions and perspectives: opportunities for oceanography from physics to biology

The evidence gathered in this study supports established theoretical and practical expectations on the sensitivity of DAS to ambient temperature. More specifically, we confirm previous observations of high-resolution ocean thermal signatures in LF-DAS data from an underwater fiber optic cable. Independent ocean water temperature signals recorded at sensors in the northern margin of the Mediterranean sea, separated from the cable by a couple of kilometers, correlate well in the long term with the LF-DAS signal at the nearest cable sections. Additional evidence of the good correlation between LF-DAS and temperature is independently provided by DAS and DSTS measurements along collocated fibers on the same cable. Furthermore, we highlight the presence of oceanic thermal anomalies with along-bottom extents of less than 1 km and up to 4 km (apparent wavelengths of hundreds of meters to a few kilometers) consistent with internal wave-induced oscillations of (sharp) ocean thermal gradients, having highly coherent propagation characteristics, apparent speeds clustered between 0.01 and 0.1 m/s, amplitudes ranging from 0.01 to more than 5 K, maximum absolute thermal rates of more than 1 K/h and periods ranging from tens of minutes and up to about the inertial period of the study region (17.5 h). The behavior of these thermal oscillations varies across the continental shelf and slope sections of the cable, pointing towards two markedly different internal wave regimes. Stable, near-inertial oscillations dominate the continental slope, while thermal stratification oscillations are evident along the shelf that are modulated on several-day scales. The clear presence of an upwelling event lasting several days on the same data, and inducing a water temperature decrease of at least 2 Kelvin at the seafloor also highlights the potential of LF-DAS for long-term underwater temperature studies.

In recent years, seismological and acoustical instrumentation has been implemented to study ocean phenomena^122–128^. DAS can be optimally generalized for these various applications, while at the same time it can provide densely sampled temperature signals across the ocean without the need for offshore campaigns, as shown in this study. With DAS measurements we may describe internal waves more systematically, i.e. their occurrence, characteristics, and better describe several aspects of their lifecycles (driving, propagation, and dissipation mechanisms). This could be leveraged to test the performance of numerical models either explicitly reproducing these processes or those at lower resolution attempting to parameterize them. This provides new experimental opportunities for oceanographic and hydrographic applications using existing telecommunications cables and other optical fiber infrastructures which, additionally to the phenomena considered in this study, could potentially be useful to study e.g. bottom temperature variability at various temporal and spatial scales (which is key for turbulence parameterizations), marine heatwaves, geothermal heat transfer across the seafloor and the response of marine ecosystems to thermal anomalies, e.g. benthic environment monitoring via combined temperature and biological (underwater acoustic) observations.

## Methods

### Instruments

The DAS interrogator unit used for our main analysis is a $\phi$-OTDR hDAS (High fidelity distributed acoustic sensor) designed by Aragón Photonics, which provides measurements in strain units. One specificity of the hDAS system is the fact that it sends a chirped light signal. Details can be found in^57,58^. The time series data sampling frequency was 100 Hz in the first couple of days of the campaign and then switched to 500 Hz.

The DSTS system used to validate the simultaneous LF-DAS (indirect) measurements was a Febus Optics G1-C set to record with a gauge length of 10 m and sampling resolution of 2.0 m over 30 km. The temporal sampling was set to 15 min to keep the data noise level at a reasonable level. The DAS system in this case was a Febus A1-R DAS interrogator with gauge length of 10 m and sampling resolution of 4.8 m over 40 km of cable. For details on the experimental setting of the LSPM cable, the reader is referred to Lior et al.^100^.

The thermistor chain used for the study is composed of a moored line equiped with a deadweight and a series of Nokalon floats, having an array of temperature sensors (HOBO Water Temp Pro v2) attached to the line. The mooring line consists of a base serving as ballast (weight of approximately 450 kg) and three ropes supported by the floats: two outer ropes forming the frame of the system and a central line supporting the temperature sensors (see Supplementary Fig. S5 for a sketch of the thermistor chain).

### Pre-processing of DAS data

Because of the high sampling rates and large DAS data volumes acquired, conventional low-pass filtering is not efficient to isolate the low-frequency content of the raw data. Thus, a multi-processing approach with a moving average was instead implemented for an optimal reduction of the thousands of channels.

Moving averages were computed for each channel using rectangular windows of 5 minutes with 60% overlap. This implies an output sampling frequency of $\sim$8.33 mHz and a maximum resolvable frequency of $\sim$1.66 mHz (the latter is the inverse of twice the averaging window size and does not match the Nyquist-criterion frequency that would be expected from the data point sampling rate because of the mismatch between the window size and its overlap). Our experience with different windows showed this combination to be a good compromise between a smoothing that is not excessive as to preserve the LF content while being enough to remove spikes, high frequency noise, and to reduce the data size by a considerable proportion.

The nearly 17 days of data were segmented in three (3) sections during acquisition due to two separate interrogator reboots. Visual inspection of the raw data shows that each of these sections has noticeable value offsets and two time gaps in-between (4 and 76 minutes each) in between (see Supplementary Fig. S6). To correct this, we demean the first time segment and adjust the remaining segments with respect to the last value of the previous ones to ensure continuity between them and to smooth out large data breaks. This “segment levelling” is performed on each channel separately. The two data gaps were filled using cubic interpolation between segments to ensure signal continuity for processing routines that require continuous time series (spectral decomposition and filtering). The resulting dataset shows good continuity, as observed from Fig. 2. The good match between the independent temperature measurements and the LF-DAS also confirms that, if existing, any instrumental drift trends are minimal and do not compromise the temperature sensing. A final pre-processing step is to remove the temporal laser noise fluctuation that is simultaneous across all channels. This was done by subtracting from the entire data ensemble the along-channel mean amplitude calculated at each time sample across a band of 200 channels dominated by background noise (i.e. standard DAS time-response or common-mode correction). For the slope section plot in Fig. 2c, which has a comparatively lower SNR than the shelf and very prominent noise peaks around 20 km along the cable, a 201-channels-long median filter was applied to each time sample separately to denoise. Frequency-filtering relied on a zero-phase order-3 Butterworth with a Tukey window at 0.01 cosine fraction pre-tapering.

The data was highpass-filtered at 0.01 mHz and tapered along both channel and time dimensions prior to frequency-wavenumber transformation with 2D Direct Fourier Transform^76^ over 16 days of data. Spectra were averaged using 13-day windows at hourly steps to increase SNR. The final frequency-wavenumber images shown in the text were max-normalized along frequency axis to highlight coherent propagation across the entire frequency range considered. The contours representing the true frequency-wavenumber spectra values were obtained after applying a Gaussian filter with 5 standard deviations along both axes to make the contours smooth and less discontinuous.

### Conversion of strain into temperature variations

As outlined in the main text, at long time scales (low frequencies), the apparent strain differences measured by DAS are expected to be caused by refractive index variations of the fiber due to temperature changes in the environment, instead of being caused by LF strain-related elongations on the fiber. Based on the phase variations induced by changes in the optical path length $\int n \textrm{d}s$ of light travelling along a longitudinal element $\textrm{d}s$ of the optical fiber, a relation describing these variations to a first-order is^38,42,46,47^:

$$\begin{aligned} \frac{\textrm{d} \varepsilon }{\textrm{d} T} = n\alpha + \frac{\textrm{d} n }{\textrm{d} T} \end{aligned}$$

where $\varepsilon ,T,n$ and $\alpha$ represent the observed (apparent) strain, the environment’s temperature, silica’s refractive index (typically around $7\cdot 10^{-6}$ K$^{-1}$ at room temperature) and its linear thermal expansion coefficient, respectively. A typical value for $\textrm{d} n/\textrm{d} T$ is known to be $10^{-5}$ (constant) while the $n\alpha$ term is expected to be much smaller, in the order of $10^{-6}$ to $10^{-7}$. Under these assumptions, a one nanostrain difference $\Delta \varepsilon$ is approximately equivalent to a temperature increase of $\Delta T \approx 0.1$ mK. In terms of relative optical phase variations $\Delta \Phi / \Phi$, the same relationship can be expressed as^39,40,47^:

$$\begin{aligned} \frac{\Delta \Phi }{\Phi } = \left( \alpha + \frac{1}{n}\frac{\textrm{d} n }{\textrm{d} T}\right) \Delta T \end{aligned}$$

An absolute normalization of each separate LF-DAS channel, i.e. between zero and the maximum value of each channel, is applied before conversion to temperature differences. Anomalously large data points corresponding to approximately $\Delta$T> 10 K were rejected.

For the comparison of LF-DAS with the thermistor chain in Fig. 2, the best-matching along-fiber channel was found via cross-correlation maxima search. The maximum correlations were found with the deepest, 50 m deep, temperature sensor of Cap Vieux, which is almost touching the seafloor. The best-matching LF-DAS channel is located $\sim$4 km away from the thermistor chain.

Pearson correlation coefficient of the multiple-day variability was found by comparison of the time series of the preprocessed LF-DAS converted to temperature changes (without filtering) with the up-sampled thermistor chain temperature signal at 50 m depth, which has a lower sampling rate. 23 consecutive channels (covering an horizontal extent of about 200 m) have Pearson correlation coefficients at or above 0.8. For the multiple-hour variability, the long-term trend of each time series was found via uniform convolution moving-average and then removed from each, so to only compare the fast variability of both. The number of samples of the uniform convolution filter was selected as the one that maximized the Pearson correlation coefficient of the fast variability signal, and was found to be equivalent to almost one day of data.

### Supplementary Information

Supplementary Information.

Supplementary Video 1.

## Data Availability

The fiber optic DSTS and the processed LF-DAS data, as well as times series used to produce Figs. 2–5, and S1, S4 and S6 are available in the following OSF repository: https://osf.io/6jf9r (https://doi.org/10.17605/OSF.IO/6JF9R). The main DAS dataset (Figs. 2,4 and S1) was recorded on the seafloor LSPM (Laboratoire Sous-marin Provence Méditerranée) cable south of Toulon, which was part of the Mediterranean Eurocentre for Underwater Sciences and Technologies (MEUST) infrastructure at the time of acquisition (see Sladen et al.^23^ for details) using an Aragón Photonics hDAS interrogator. MEUST is financed with the support of the CNRSIN2P3, the Region Sud, France (CPER the State (DRRT), and FEDER. Auxiliary DAS and DSTS datasets were recorded on the same cable using a Febus Optics G1-C and a Febus A1-R interrogators, respectively. The latter were used to produce Figs. 5 and S4. Bathymetry data of the study region (South of France/Gulf of Lions) to produce Fig. 1 was freely available from SHOM^129^ and can be accessed here: https://diffusion.shom.fr/pro/mnt-facade-gdl-ca-homonim.html. The map was produced with QGIS v3.22 (QGIS.org, 2022. QGIS Geographic Information System. QGIS Association). The data of the thermistor chain of Cap Vieux is provided for free by Sartoretto et al.^60^ (https://doi.org/10.17882/86522) and can be retrieved upon request (Parameters: Toulon_(CapSicie), 2019, All Depths) from the regional temperature observation network (T-MEDNet), https://t-mednet.org/request-data?view=tdatarequest&site_id=38. AROME operational atmospheric model data was obtained from Météo-France (https://donneespubliques.meteofrance.fr/?fond=produit&id_produit=131&id_rubrique=51). The sea surface temperature model data to produce Supplementary Video S1 was obtained from https://marc.ifremer.fr/en/results/temperature_and_salinity/mediterranean_model^83^. Technical details at https://forms.ifremer.fr/lops-oc/marc-f2-mars3d/. FES2014 sea level and tidal current model data^130^ used to generate Fig. S2 is openly available upon request from Aviso+ (https://www.aviso.altimetry.fr/). Data processing and analyses largely relied on standard Python libraries, e.g. SciPy (https://scipy.org/), NumPy (https://numpy.org/), Pandas (https://pandas.pydata.org/), Matplotlib (https://matplotlib.org/), h5Py (https://www.h5py.org/); plus dedicated libraries for optimization: Dask^131^; seismic data processing: ObsPy^132^; and additional specialized libraries: Sklearn^133^; EMD^70^ and cmocean^134^.
