# Supplementary material for: High resolution seafloor thermometry for internal wave and upwelling monitoring using Distributed Acoustic Sensing
Source: Sci Rep. 2023 Oct 14;13:17459. doi: 10.1038/s41598-023-44635-0 (PMC10576814; doi:10.1038/s41598-023-44635-0)
Supplement: Supplementary file 1 — Supplementary Information. [file 41598_2023_44635_MOESM1_ESM.pdf]

Supporting Information for "High resolution seafloor thermometry for internal wave and upwelling monitoring using Distributed Acoustic Sensing"

Julián Pelaez Quiñones[1,*], Anthony Sladen[2], Aurelien Ponte[2], Itzhak Lior[3], Jean-Paul Ampuero[1], Diane Rivet[1], Samuel Meulé[4], Frédéric Bouchette[4], Ivane Pairaud[2], and Paschal Coyle[5]

[1]Université Côte d'Azur, CNRS, Observatoire de la Côte d'Azur, IRD, Géoazur, Sophia Antipolis, *250* rue Albert Einstein, *06560*, Valbonne, France
[2]IFREMER, Université de Brest, CNRS, IRD, Laboratoire d'Océanographie Physique et Spatiale, IUEM, Brest, France
[3]Institute of Earth Sciences, The Hebrew University, Jerusalem, Israel
[4]Geosciences-M/GLADYS, Université de Montpellier, CNRS, Montpellier, France
[5]Aix-Marseille Université, CNRS/IN2P3, CPPM, Marseille, France
[*]julian.pelaez@geoazur.unice.fr

Contents of this file

- Texts S1 to S4:
    1. Some basics of Distributed Acoustic Sensing
    2. Inertial variability
    3. Empirical Mode Decomposition and Hilbert-Huang Transform parameters
    4. Estimation of tidal current-induced temperature variations over a sloping bottom
- Figures S1 to S6
    1. Sample Intrinsic Mode Functions (IMFs)
    2. Estimated tidal-current induced temperature anomalies
    3. Schematic interpretation of LF-DAS thermal features
    4. Collocated DSTS and LF-DAS measurements
    5. Sketch of thermistor chain structure
    6. Illustration of LF-DAS pre-processing

Introduction

This file contains complementary information to our main manuscript, principally details about the sensing instruments, methods, processing and some additional figures.

Text S1. Some basics of Distributed Acoustic Sensing

Distributed Acoustic Sensing (DAS) systems make use of single optic fibers cased inside (un)armored cables, for instance existing Telecommunication cables, to sense the environment. Currently, most DAS systems require a dark fiber to operate, and can provide useful data at maximum distances along cable of up to one or two hundred kilometers, although recent breakthroughs include successful DAS measurements along cable simultaneously data transmission and measurements of up to one thousand kilometers [1, 2, 3]. Coherent laser pulses are regularly sent along the fiber and their Rayleigh back-scattered signature due to imperfections of the fiber are used as a proxy for temperature and strain perturbations affecting the optical path length (due to local elongations and refractive index variations of the fiber) over specific sections of the cable [4, 5]. These perturbations can be traced-back along specific locations along the fiber by converting the two-way travel time of the back-scattered light to distances with the known speed of light in silica. Measurements are averaged along a few meters of cable (gauge length) at a defined distance step (spatial sampling). In contrast to DAS, Distributed Temperature Sensing (DTS) is based on Raman-scattering, while Distributed Temperature and Strain Sensing (DSTS) is based on Brillouin-scattering.

Text S2. Inertial variability

The inertial period T_c at a given latitude θ reflects the variability time scale of important mesoscale to large scale oceanographic processes. This period is determined by the Coriolis effect due to the angular momentum conservation for traveling objects that are subjected to the earth's rotation centrifugal force and can be estimated via:

$$T_c = f_c^{-1} = (2\Omega \sin\theta)^{-1}$$

where f_c is known as the Coriolis frequency and Ω is the rotation rate of the earth ($\sim 7.29 \times 10^{-5}$ rad/s) [6, 7, 8, 9]. This translates into an inertial period of ~17.5h at the mean latitude of the Toulon cable (43°N).

Text S3. Empirical Mode Decomposition and Hilbert-Huang Transform parameters

EMD and HHT analyses [10] were performed by using the EMD Python package [11]. Several of the examples and built-in functions of the package were implemented in our study. The mask sifting (mode separation) scheme [12] produced the best results for the LF-DAS data. This approach allows us to obtain a set of well-behaved Intrinsic Mode Functions (IMFs) that represent generalized spectral components or empirical modes of the input signal. A proper sifting leads to IMFs that are purely oscillatory functions with zero reference levels from which instantaneous amplitude and frequency attributes are obtained by means of a Hilbert-Huang Transform (HHT) [13]. The masks are monochromatic signals introduced into the Intrinsic Mode Function (IMF) under consideration to avoid mixing of modes with very different frequencies: as the high frequency components are always captured and separated first during the sifting, a mask signal with a frequency higher than a long period oscillation in the signal contributes to separate the latter correctly from the other higher frequency components. Most of the default mask sifting parameters of the package were the basis of our processing. The amplitude of these masks were uniformly computed as ratios of the standard deviation of the input for all IMFs; their frequency successively increasing at factors of 2. Four masks were applied to each IMF and the sift threshold was set to 10^{-8}. Eight IMFs were calculated in total.

The instantaneous attributes (amplitude and frequency) of each IMF were found via amplitude-normalized Hilbert transform (NHT) as in [14]. Channels with anomalous extrema were muted under a 3-standard deviation outlier criterium. We applied a logarithmic binning of 1000 grid points between 0.001 and 1.0 mHz to ensure enough spectral resolution. Amplitudes were stacked to obtain the binned HHT. The HHT spectra were normalized as power spectral density (divided by $f_{sampling} \cdot N_{samples}$). To obtain the HHT spectra, we averaged all the instantaneous attributes of each IMF over a selected range of channels. This results in a stacked spectrogram-like output representing the dominant spectral power spectral density over a section of cable. The LF-DAS time series were pre-filtered with a highpass at 0.0007 mHz (equivalent to nearly 16 days - the total duration of the deployment) and pre-averaged every two consecutive channels to increase their SNR. The final images were smoothed using a Gaussian kernel convolution filter with one standard deviation. For Figs 3b,c, each IMF is weighted by its instantaneous amplitude, so to obtain an image analogous to a spectrogram that captures the time-evolution of the spectral components.

Care was taken to select a timespan for analysis with no large data breaks and to reject channels with anomalously uniform or large values or spikes (as seen from Fig. 2a,b), as these artifacts can largely affect the EMD [15]. Furthermore, the averaging

of the instantaneous attributes of each IMFs across a sufficiently long cable range helps to balance out such undesired effects, in case that artifacts may remain at some channels. Orthogonality tests [10] and Pseudo Mode Mixing indices [16] were computed for the IMFs of a few channels to check for consistency of the results and positive results were obtained. Some instantaneous frequency overlap between the instantaneous frequencies of some adjacent IMFs as well as sporadic negative instantaneous frequency values still exist in the output HHT, but these contributions remain low in overall and were significantly suppressed after channel averaging. Supplementary Figure S1 shows an example of such decomposition for a selected channel using the EMD Python package.

Text S4. Estimation of tidal current-induced temperature variations over a sloping bottom

The vertical current w associated with an horizontal current u flowing against bathymetry h is:

$$w = u \times \partial_x h,$$

where ∂_x denotes a horizontal gradient (slope) in radians. The vertical displacement η associated with a periodic, sinusoidal vertical velocity of magnitude w and angular frequency ω is: $\eta = w/\omega$. Using semi-diurnal and/or diurnal frequencies, i.e. $\omega = 1.4 \times 10^{-4}$ and/or $\omega = 7.2 \times 10^{-5}$ rad/s, respectively, one can estimate the local variation of temperature δT associated with a mean vertical thermal gradient $\partial_z T$ as:

$$\delta T = \eta \times \partial_z T$$

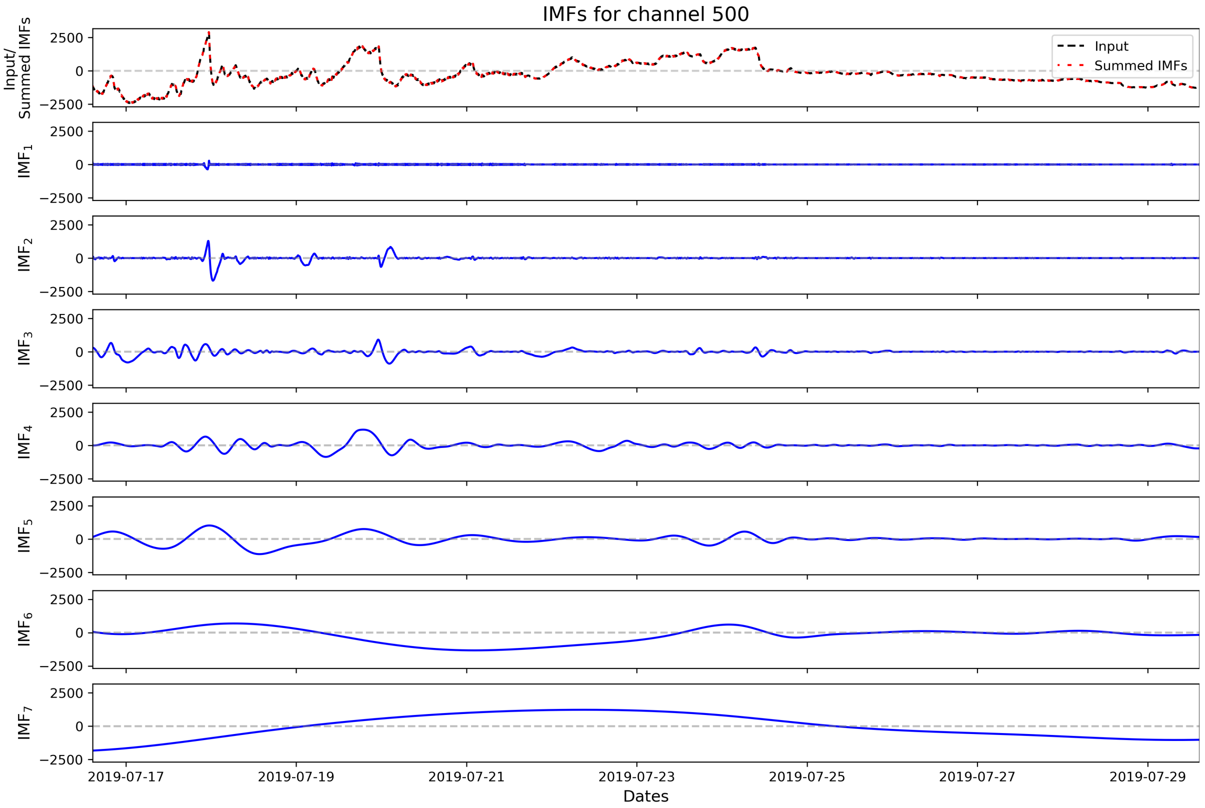

Figure S1. Sample Intrinsic Mode Functions (IMFs) for a selected LF-DAS channel.

References

1. Marin, J. M. *et al.* Simultaneous Distributed Acoustic Sensing and communication over a two-mode fiber. *Opt. Lett.* **47**, 6321–6324, DOI: 10.1364/OL.473502 (2022).

2. He, H. *et al.* Integrated sensing and communication in an optical fibre. *Light. Sci Appl* **12**, 265–273, DOI: 10.1038/s41377-022-01067-1 (2023).

3. Ip, E. *et al.* DAS Over 1,007-km Hybrid Link With 10-Tb/s DP-16QAM Co-Propagation Using Frequency-Diverse Chirped Pulses. *J. Light. Technol.* **41**, 1077–1086, DOI: 10.1109/JLT.2022.3219369 (2023).

4. López-Higuera, J. M. (ed.) *Handbook of Optical Fibre Sensing Technology* (John Wiley & Sons, ltd, 2002), 1 edn.

5. Hartog, A. *An Introduction to Distributed Optical Fibre Sensors* (CRC Press, 2017), 1 edn.

6. Tintoré, J., Wang, D.-P., Garćia, E. & Viúdez, A. Near-inertial motions in the coastal ocean. *J. Mar. Syst.* **6**, 301–312, DOI: 10.1016/0924-7963(94)00030-F (1995).

7. Gerkema, T. & Zimmerman, J. An introduction to internal waves. *Lect. notes, Royal NIOZ, Texel, 2008* (2008).

8. Miropol'sky, Y. & Shishkina, O. *Dynamics of Internal Gravity Waves in the Ocean*. Atmospheric and Oceanographic Sciences Library (Springer Netherlands, 2013).

9. Massel, S. *Internal Gravity Waves in the Shallow Seas* (Springer Cham, 2015), 1 edn.

10. Huang, N. E. *et al.* The empirical mode decomposition and the Hilbert spectrum for nonlinear and non-stationary time series analysis. *Proc. R. Soc. Lond. A.* **454**, 903–995, DOI: 10.1038/s41598-020-72193-2 (1998).

11. Quinn, A. J., Lopes-dos Santos, V., Dupret, D., Nobre, A. C. & Woolrich, M. W. EMD: Empirical Mode Decomposition and Hilbert-Huang Spectral Analyses in Python. *J. Open Source Softw.* **6**, 2977, DOI: 10.21105/joss.02977 (2021).

12. Deering, R. & Kaiser, J. The use of a masking signal to improve empirical mode decomposition. In *Proceedings. (ICASSP '05). IEEE International Conference on Acoustics, Speech, and Signal Processing, 2005.*, vol. 4, iv/485–iv/488 Vol. 4, DOI: 10.1109/ICASSP.2005.1416051 (2005).

13. Huang, N. E. & Wu, Z. A review on Hilbert-Huang transform: Method and its applications to geophysical studies. *Rev. Geophys.* **46**, DOI: 10.1029/2007RG000228 (2008).

14. Huang, N. E. *et al.* On instantaneous frequency. *Adv. Adapt. Data Analysis* **01**, 177–229, DOI: 10.1142/S1793536909000096 (2009).

15. Stallone, A., Cicone, A. & Materassi, M. New insights and best practices for the successful use of empirical mode decomposition, iterative filtering and derived algorithms. *Sci Rep* **10**, DOI: 10.1038/s41598-020-72193-2 (2020).

16. Wang, Y.-H., Hu, K. & Lo, M.-T. Uniform phase empirical mode decomposition: An optimal hybridization of masking signal and ensemble approaches. *IEEE Access* **6**, 34819–34833, DOI: 10.1109/ACCESS.2018.2847634 (2018).

17. Lucas, A. J. & Pinkel, R. Observations of coherent transverse wakes in shoaling nonlinear internal waves. *J. Phys. Oceanogr.* **52**, 1277 – 1293, DOI: 10.1175/JPO-D-21-0059.1 (2022).

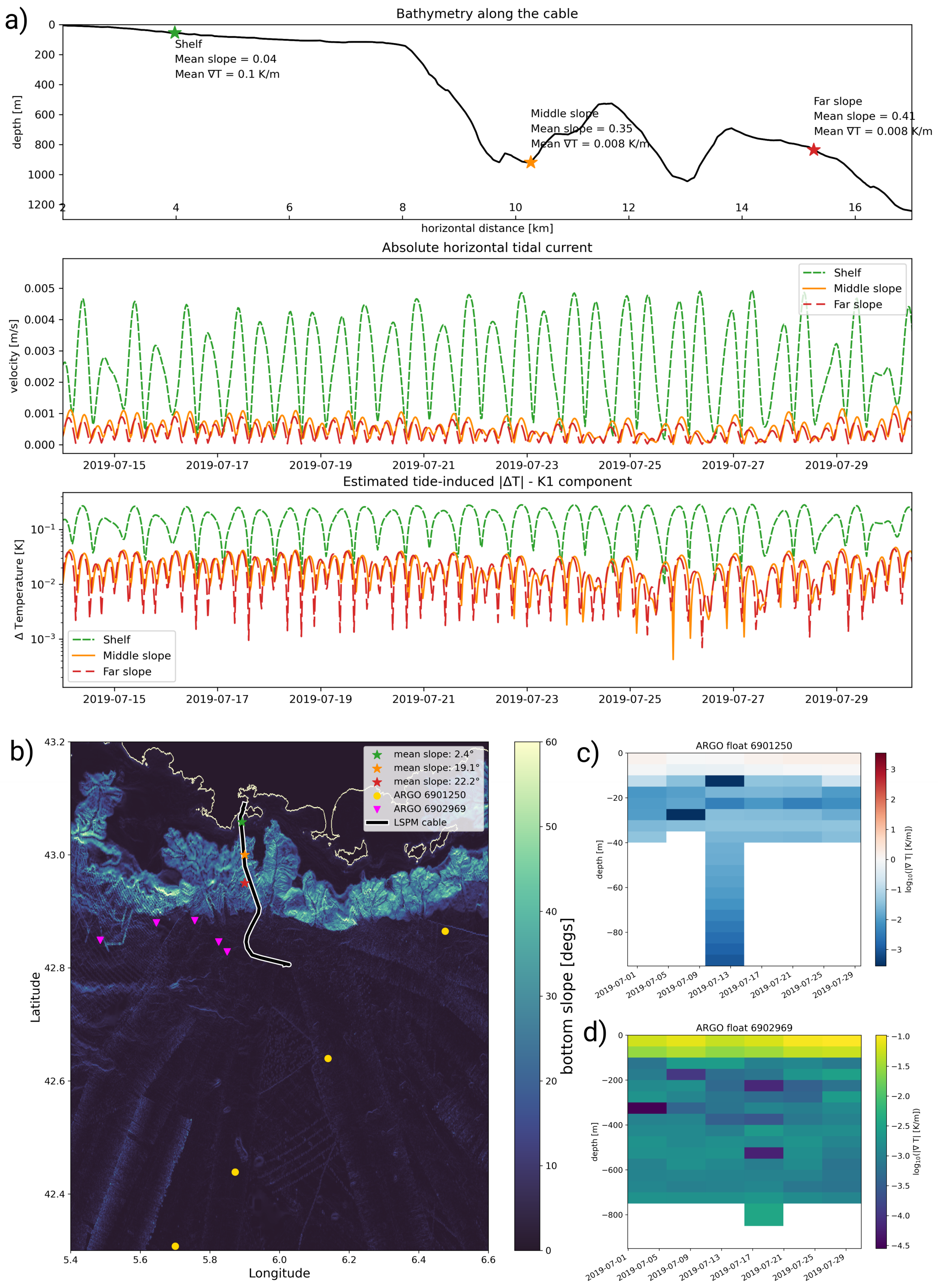

Figure S2. a) Bathymetry along the cable and the estimated tidal-current induced temperature anomalies derived from the horizontal tidal current components (FES2014 model) at the three highlighted locations. The results are valid for the K1 tidal component ($\sim$24 h), while values for the main semidiurnal components are expected to be as half as large. b) Bathymetric slope map with the markers indicating the location of the three tidal model points and that of the available ARGO floats in c) and d), which were used to estimate the mean thermal gradient over the continental slope points (taken from the deepest half of d)). Mean slopes were estimated on 2x2 km cells. The mean thermal gradient over the shelf was estimated from the deepest 30 m of the thermistor chain at Cap Vieux (Figs. 1 and 2 in main text) during the extent of the experiment. The derivation method is as explained in Text S4.

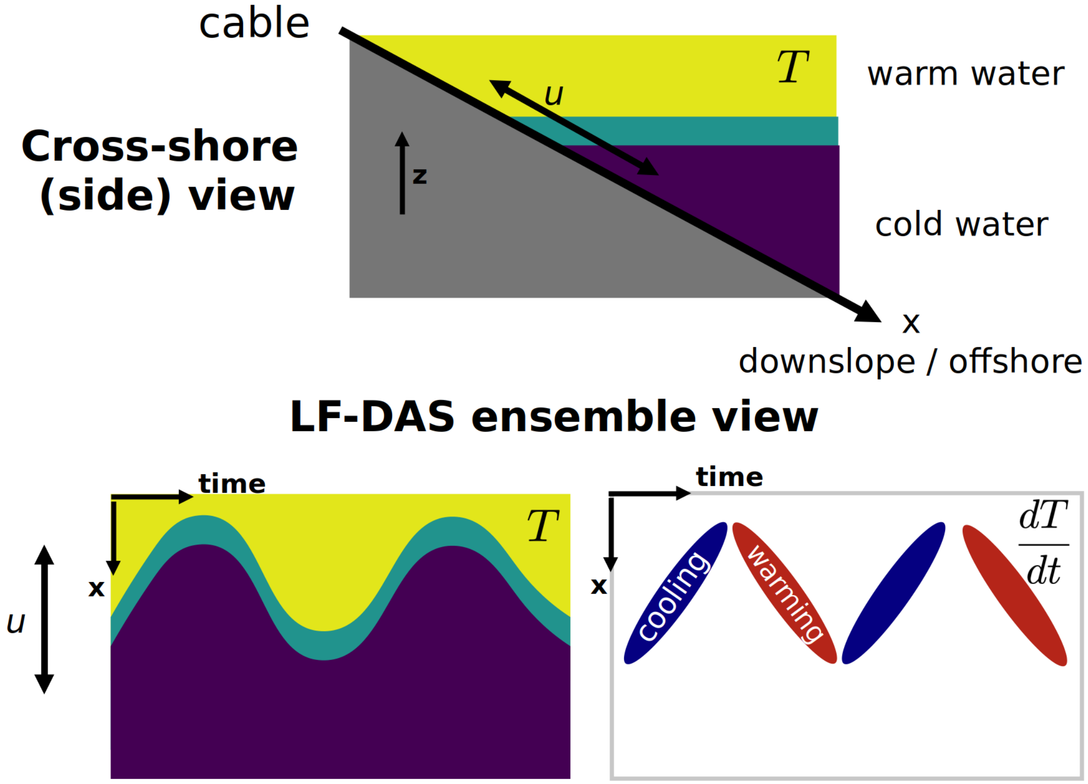

Figure S3. Schematic interpretation of the "V"-shaped thermal features observed in LF-DAS, assuming a vertical thermal gradient that is monotonically advected, as in Fig. 4 in Lucas & Pinkel (2022) [17]. Top: Idealized water column with a sharp, localized gradient (i.e. thermocline) separating cold water at depth and warm water above. Bottom-left: temperature signal along the cable produced by successive downslope/offshore and upslope/onshore oscillating current u (e.g. prompted by a monochromatic internal wave) brings warm water offshore (positive x) and cold water onshore, respectively. Bottom-right: the associated temperature time rate of change produces repeating "V"-shaped patterns with cooling branches pointing onshore and warming branches propagating offshore. The same process could occur due to along-slope displacements of an horizontal temperature gradient albeit with no preferred orientation of the cooling and warming branches given the equal probability, in general, of encountering horizontal temperature gradients of either sign.

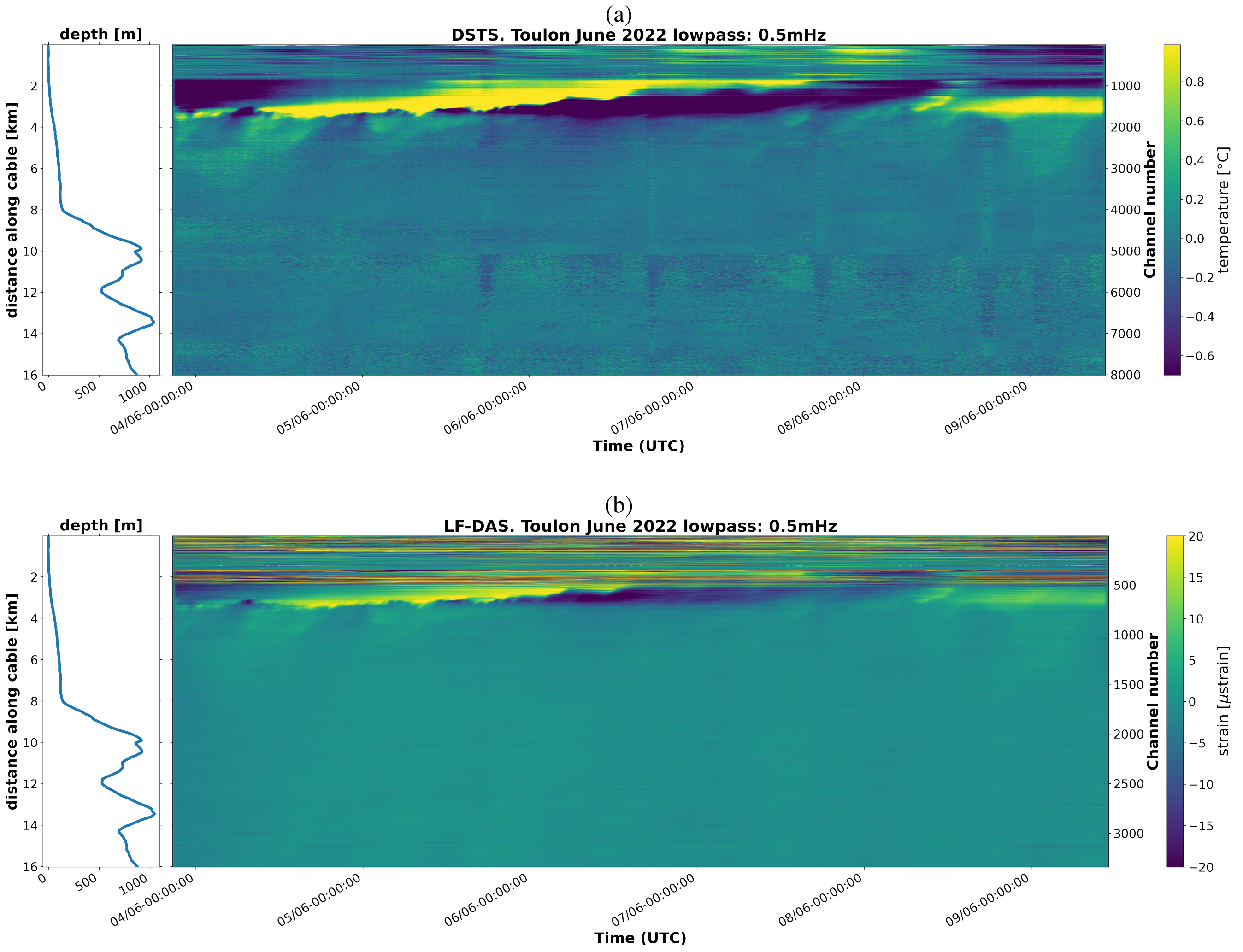

Figure S4. Collocated DSTS and LF-DAS measurements in Toulon, June 2022 - Filtered ensemble comparison. Lowpassed DSTS (a) and LF-DAS (b)

(c)

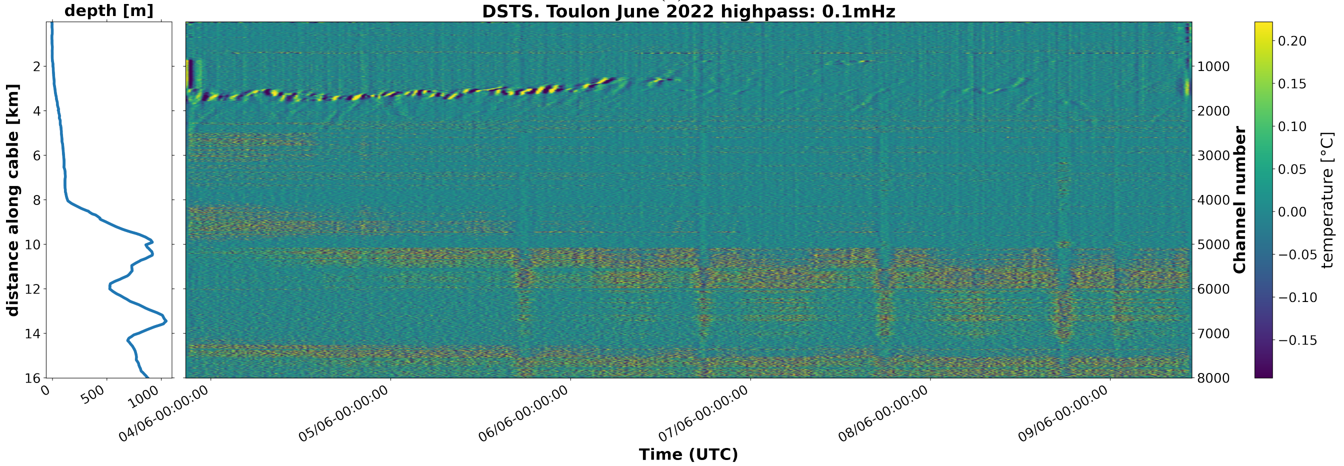

(d)

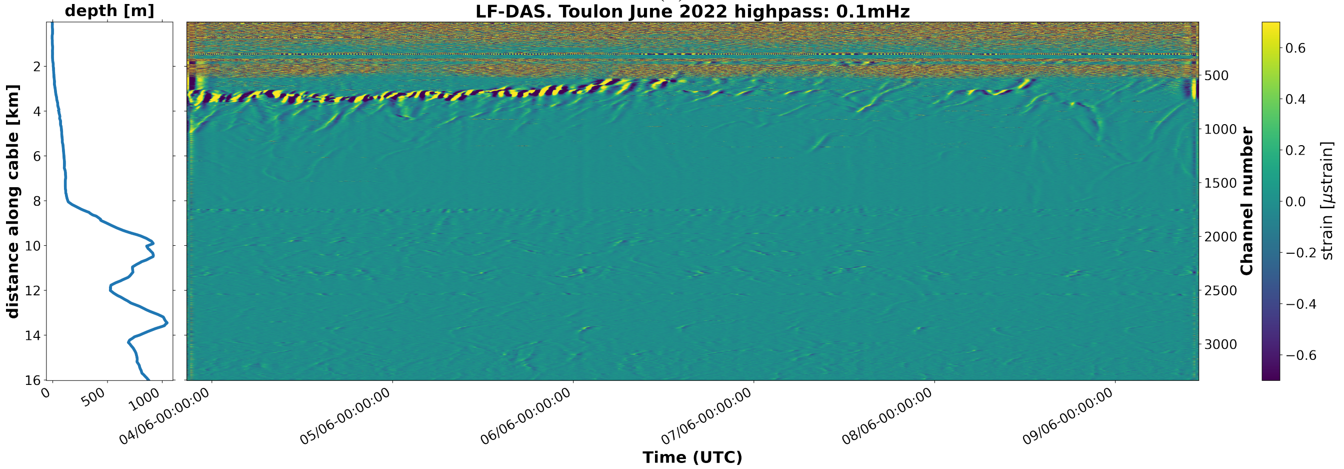

Figure S4. (cont.) collocated DSTS and LF-DAS measurements in Toulon, June 2022 - Filtered ensemble comparison. Highpassed DSTS (c) and LF-DAS (d).

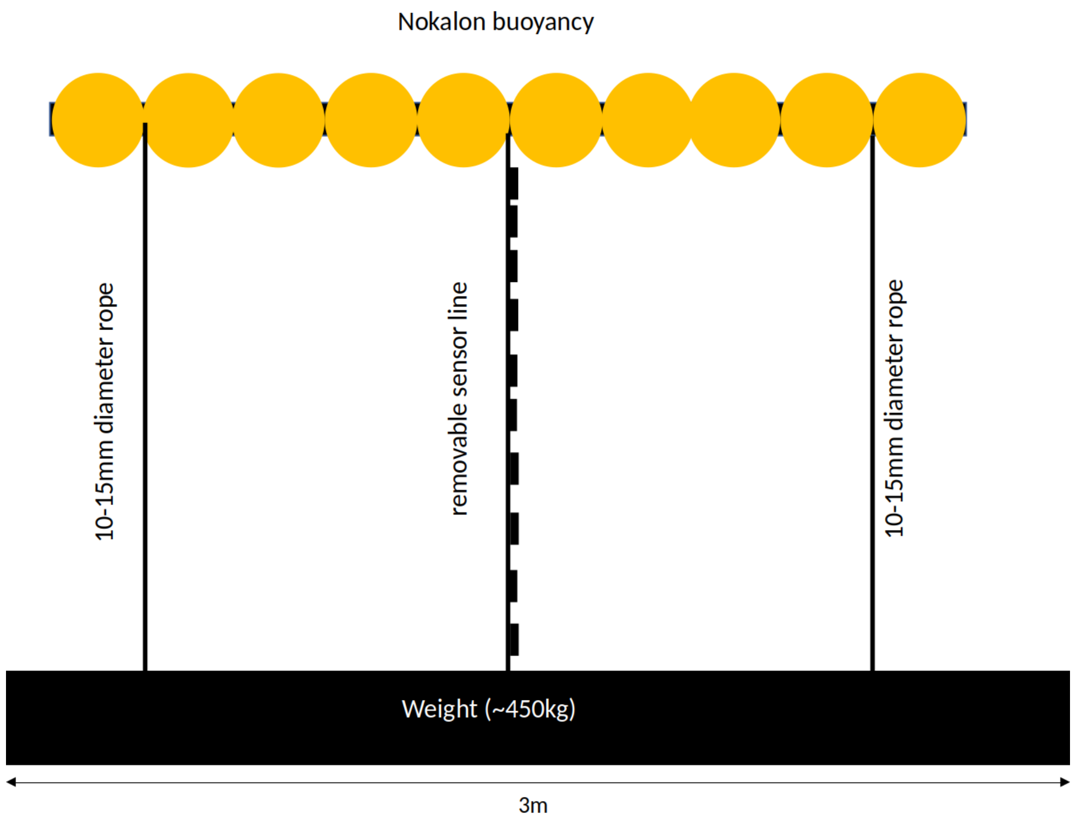

Figure S5. A sketch of the structure of the TMEDNet thermistor chain used in this study (originally in french, property of the manufacturer).

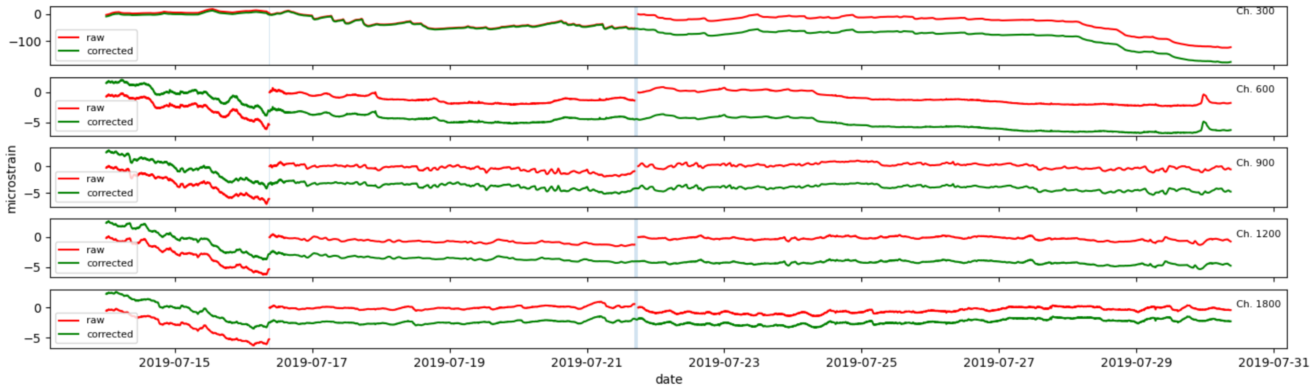

Figure S6. Selected DAS channels before (red) and after (green) segment leveling and gap interpolation. The light blue shaded areas mark the location of the two gaps in the data. The timespan corresponds to that presented in Fig. 2 of the main text.
